# A 32-Channel Sleeve Antenna Receiver Array for Human Head MRI Applications at 10.5 T

**DOI:** 10.1109/TMI.2023.3261922

**Published:** 2023-08-31

**Authors:** Myung Kyun Woo, Lance Delabarre, Matt Waks, Russell Lagore, Jeehoon Kim, Steve Jungst, Yigitcan Eryaman, Kamil Ugurbil, Gregor Adriany

**Affiliations:** Division of Biomedical Engineering, Hankuk University of Foreign Studies, Yongin 17035, Republic of Korea; Center for Magnetic Resonance Research (CMRR), University of Minnesota, Minneapolis, MN 55455 USA;; Center for Magnetic Resonance Research (CMRR), University of Minnesota, Minneapolis, MN 55455 USA; Center for Magnetic Resonance Research (CMRR), University of Minnesota, Minneapolis, MN 55455 USA; Center for Magnetic Resonance Research (CMRR), University of Minnesota, Minneapolis, MN 55455 USA; Department of Electrical Engineering, Case Western Reserve University, Cleveland, OH 44106 USA; Program of Advanced Musculoskeletal Imaging (PAMI), Cleveland Clinic, Cleveland, OH 44196 USA; Center for Magnetic Resonance Research (CMRR), University of Minnesota, Minneapolis, MN 55455 USA; Center for Magnetic Resonance Research (CMRR), University of Minnesota, Minneapolis, MN 55455 USA; Center for Magnetic Resonance Research (CMRR), University of Minnesota, Minneapolis, MN 55455 USA; Center for Magnetic Resonance Research (CMRR), University of Minnesota, Minneapolis, MN 55455 USA

**Keywords:** Dipole antenna, magnetic resonance imaging, radiofrequency coil, receiver array, sleeve antenna, ultra-high field

## Abstract

For human brain magnetic resonance imaging (MRI), high channel count (≥32) radiofrequency receiver coil arrays are utilized to achieve maximum signal-to-noise ratio (SNR) and to accelerate parallel imaging techniques. With ultra-high field (UHF) MRI at 7 tesla (T) and higher, dipole antenna arrays have been shown to generate high SNR in the deep regions of the brain, however the array elements exhibit increased electromagnetic coupling with one another, making array construction more difficult with the increasing number of elements. Compared to a classical dipole antenna array, a sleeve antenna array incorporates the coaxial ground into the feed-point, resulting in a modified asymmetric antenna structure with improved intra-element decoupling. Here, we extended our previous 16-channel sleeve transceiver work and developed a 32-channel azimuthally arranged sleeve antenna receive–only array for 10.5 T human brain imaging. We experimentally compared the achievable SNR of the sleeve antenna array at 10.5 T to a more traditional 32-channel loop array bult onto a human head-shaped former. The results obtained with a head shaped phantom clearly demonstrated that peripheral intrinsic SNR can be significantly improved compared to a loop array with the same number of elements – except for the superior part of the phantom where sleeve antenna elements are not located.

## I. INTRODUCTION

Development of ultra-high field (UHF) magnetic resonance imaging (MRI) is foremost motivated by the important benefit of increased signal-to-noise ratio (SNR) [1], [2], [3], [4] that comes with higher magnetic field strength, and which is required to support increased resolution with human brain imaging. The demand of UHF MRI has been increased particularly for functional MRI (fMRI) [5], [6], [7] - the premier methodology which allows exploration of functional whole brain networks - as well as diffusion tensor imaging (DTI) [6], [8] to study cortical connectivity. These informative human biological and physiological MRI acquisition techniques have become increasingly more efficient with the higher SNR produced from radiofrequency (RF) arrays optimized with higher channel counts (e.g. 32). The associated spatially distinct sensitivity profiles of each array element support increased parallel imaging acceleration [9] and improved parallel imaging performance [10], [11].

Recently, the transformation of RF coil designs at UHF from the strict near field domain to far field antenna concepts have been investigated [12], [13]. At UHF, such as 10.5 tesla (T) [14], [15], [16], [17], [18], the associated RF wavelength begins to become smaller in tissue [6], [7], [19], [20] than that of the target anatomy. This can cause significant RF B^+^_1_ field (defined as the transmitted magnetic (B) field generated by RF coils) variations in biological tissue and can lead the increased imaging inhomogeneity [20], [21]. At UHF, radiative type antennas (e.g. dipole) have shown promising performance with deeper penetration depth not only for transmit coils [13], [22], [23], [24], [25], but also for receive coils [16], [26], [27], [28], [29]. Radiative antenna arrays have the ability to accommodate deeper reception compared to traditional loop arrays. High channel count dipole antenna arrays have been proposed and reported [27], [29]. However, dipole antennas and their coaxial feed cables (typically routed in parallel with one leg of the dipole antenna) can cause detrimental interactions in practice, particularly for head applications [17], [30]. This is further exaggerated at UHF for receive arrays, where the coaxial cables associated with a receive coil feed point can interact with a transmit coil. The resulting interaction among a transmit coil, a receive coil, and coaxial cables can generate complex field interference which cannot be easily solved by the use of balanced to unbalanced (balun) matching techniques, nor with cable traps [31], [32], [33]. Unfortunately, such interaction between the transmitter and the coaxial cables in an array can change field patterns and degrade the antenna performance [34], [35].

Naturally, radiative antenna arrays present greater challenges in minimizing the mutual coupling between neighboring elements [36], [37], [38], [39] and consequently for high channel count arrays more robust decoupling approaches need to be developed. In our previous publication [40], we introduced a sleeve antenna concept for a transceiver array where we demonstrated improved element decoupling and cable interference without losing antenna efficiency. With the same dimensions and number of channels, the 8-channel sleeve antenna array (Min: −14.5 dB and Max: −19.6 dB) shows lower S_21_ values compared to the 8-channel loop array (Min: −9.5 dB and Max: −16 dB) [17]. Also, the sleeve antenna concept with the end-fed structure shows a practical advantage due to the coaxial cable position which is collinearly aligned with the sleeves [40]. This implies that a sleeve antenna receiver array has a potential for reduced interference between the coaxial receive cables and the transmitter. To reduce interaction in our previous publication, a sleeve antenna was designed utilizing a preamplifier decoupling technique which added an additional 15 dB decoupling improvement [41].

With these prior established base technologies, we here extend the concept further and propose the development of a 32-channel sleeve antenna receive-only array for UHF MRI applications. For the comparison to a prior described 10.5 T 32-channel loop receiver, we built the prototype 32-channel receive-only sleeve antenna array on an elliptical former similar in size to the loop head arrays [42]. We measured the noise covariance matrix as well as individual receive fields experimentally. We also experimentally compared intrinsic signal-to-noise ratio (iSNR) and g-factor maps of the sleeve antenna array to those of a 32-channel loop receive array [42] with a similar housing dimension at 10.5 T.

## II. Methods

### A. Construction of a 32-Channel Sleeve Antenna Receiver Array

The overall resonant length of a sleeve antenna array element [40], [43], [44] is similar to a dipole antenna with some key variations. Most significantly, the primary imaging conductor of the sleeve antenna is configured as a ‘monopole antenna’ built from the center conductor of a coaxial cable with a ‘sleeve’, which is basically a cable trap, placed over the shield of the feed cable [43], [45]. The length of this monopole part of the sleeve antenna can be adjusted independently but combined with the sleeve part requires lamda (λ)/2 resonance length. This way with a sleeve antenna, the current distribution can be slightly modified depending on the ratio of the length between the monopole antenna and the sleeve [40], [45], [46].

**Fig. 1.**
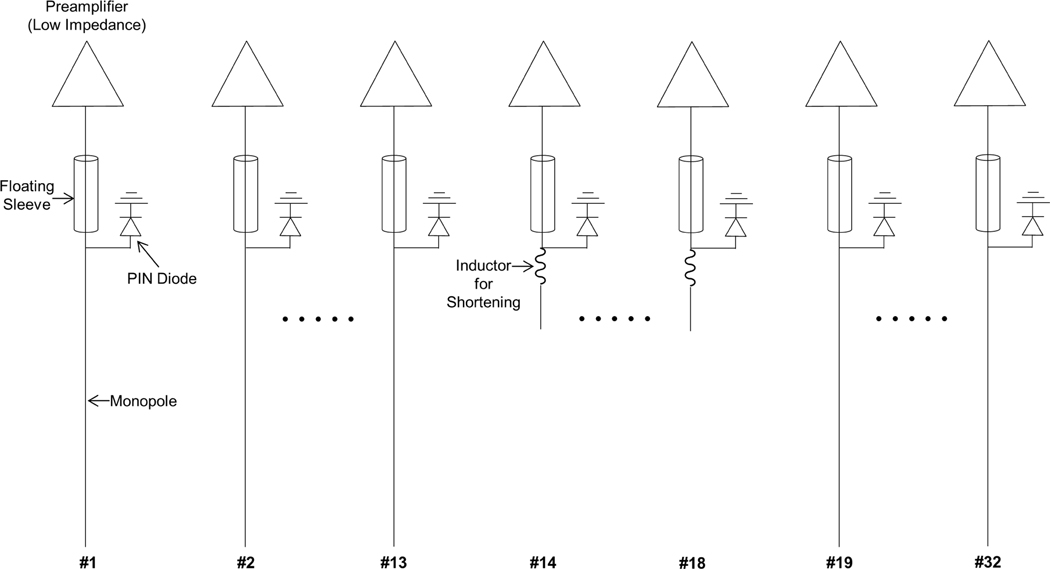
Schema of a 32-channel sleeve antenna receiver array. Channel #14 to #18 were shortened with inductors to reduce the physical length of antennas on the anterior side of the array. Coupling among all 32-channel sleeve antennas was reduced by the asymmetric structure of each antenna which has higher impedance compared to the symmetric structure of antennas. More distal coil interactions among the next nearest neighbors were reduced by preamplifer decoupling using low input impedance preamplifers.

In Fig. 1, 27 channels of the sleeve antenna array are configured with a 19 cm long monopole antenna, a 5 cm floating sleeve, and a 12 cm long coaxial cable which passes through a floating sleeve. The length of each individual monopole antenna channel was adjusted ± 0.5 cm for fine tuning. To support task presentation and allow for an opening in front of the eyes, these 27 channels are combined for the five shorter (5 cm) channels with integrated inductors (475 nH) for resonance positioned over the forehead. Each channel of the resulting 32-channel sleeve antenna array was then connected to a low-input impedance preamplifier (WanTcom, Chanhassen, MN, USA). One shunt PIN diode was placed in each circuit from the center conductor to the ground for the detuning of the receiver array during B_1_ transmission.

The sleeve antenna receiver array was mounted on a polyethylene terephthalate glycol-modified (PETG) housing which was fabricated using a 3D printer (F410, Fusion3 Design, Greensboro, NC, USA). This PETG former was configured into three sections: monopoles (19 cm), sleeves and coaxial cables (12 cm), and lastly the preamplifiers and connections to the Siemens coil plug (2 cm). All channels of the 32-channel sleeve antenna array were located directly on the 3D printed former. The dimension of the sleeve antenna array has a minor axis of 19 cm and major axis of 22.6 cm with azimuthally arranged 32 individual channels tightly spaced at 1.5 cm apart. Floating cable traps were used for each sleeve antenna element [33].

The cylindrically shaped floating cable trap has 1.2 cm outer diameter and 0.5 cm inner diameter which accommodates a K02252-D coaxial cable (Huber Suhner, Herisau, Switzerland). Two high Q ceramic chip capacitors (100B series, American Technical Ceramics, Huntington Station, NY, USA) and one variable capacitor (GSX366, Sprague-Goodman, Westbury, NY, USA) were used to adjust the trap resonance frequency.

### B. A 32-Channel Loop Receiver Array for SNR Comparison and a 16-Channel Loop Transmitter Array

**Fig. 2.**
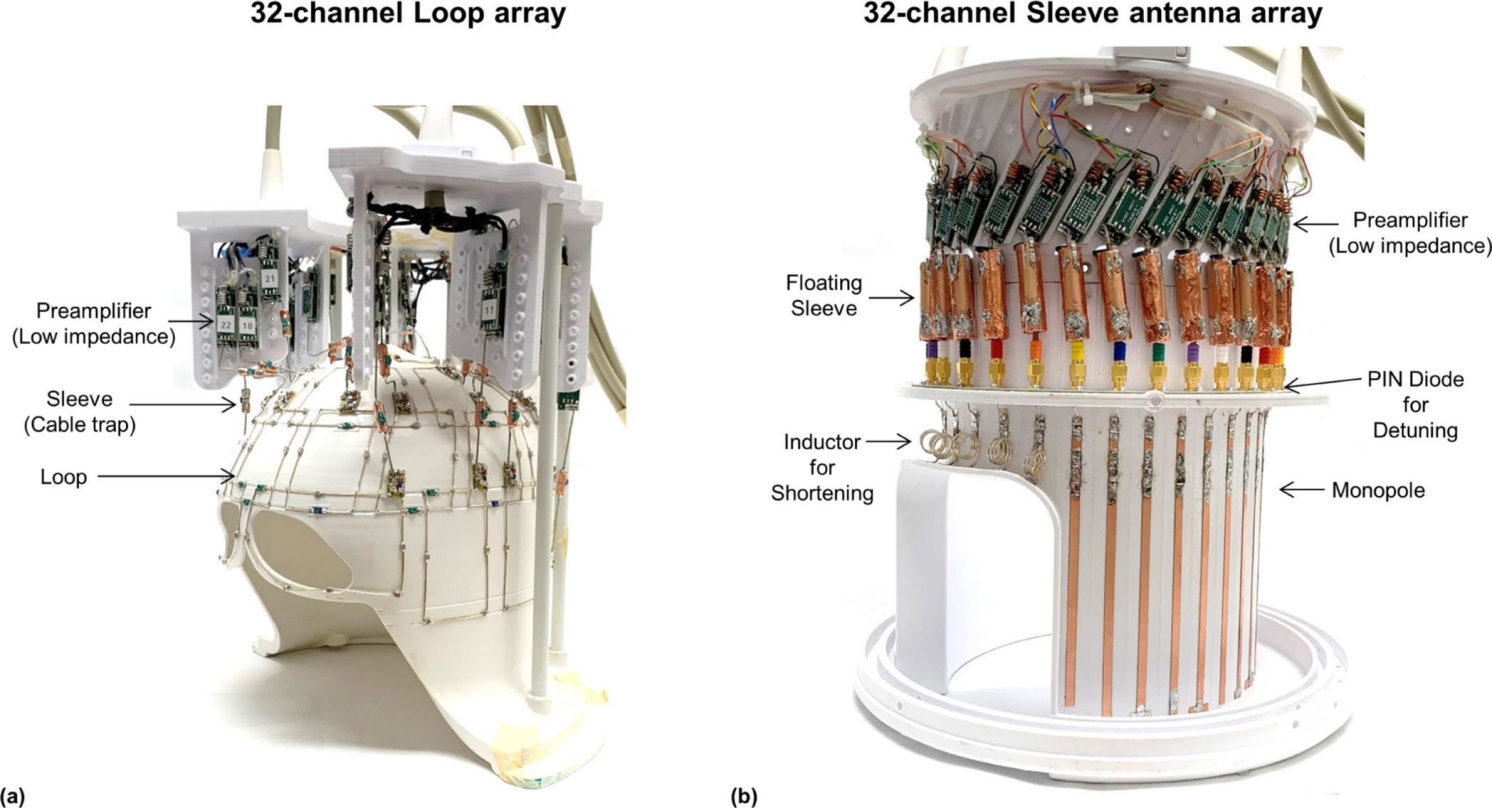
Photographs of the 32-channel loop (a) and sleeve antenna (b) arrays.

For iSNR evaluation, the 32-channel sleeve antenna array (Fig. 2b) was compared to a 32-channel loop array (Fig. 2a) [42] mounted on a close-fitting 3D printed head-shaped former with similar overall inner dimensions (inner diameter ~19 cm) but with a conformal fit to the top of the head. The loop array also utilized preamplifier decoupling in order to reduce intraelement interactions. The loops were rectangularly shaped and arranged in four rows along the z-axis. For further decoupling, neighboring channels in z-direction were overlapped whereas neighboring channels in x-y direction were self-decoupled using a previous technique [39]. One clover leaf shaped loop coil was located at the top of the former to cover the superior part of the brain.

A home-built 16-channel loop array was used as the transmitter for both the 32-channel loop and sleeve antenna receiver arrays [47]. This 16-channel transmit array consisted of sixteen 10 × 10 cm^2^ loop coils arranged in two rows of eight channels. The 3D printed conformal former supported the inner receiver (32-channel loop and sleeve antenna arrays) insert. To tune the 16-channel loop transmitter at 10.5 T, each loop was built with 15 distributed ceramic chip capacitors with a non-overlapping coil layout. Inductive decoupling was used between the nearest neighbors. The 16-channel loop transmitter was detuned using PIN diodes during signal reception. During the transmit phase, this loop transmitter was fully tuned and resonant.

### C. Experimental Setup

All detuning states and preamplifier decoupling levels were measured and optimized using a 16-channel vector network analyzer (ZNBT8, Rohde & Schwarz, Munich, Germany) with a human head shaped phantom (diameter: 15 cm and height: 18 cm). This polyvinylpyrrolidone-agar gel (*σ* of 0.69 S/m and *ε*_r_ of 49) phantom was used to acquire all of the experimental data sets. Dielectric parameters were measured with a DAKS-12 coaxial dielectric probe (SPEAG, Zurich, Switzerland) at 447 MHz. A small cylinder (diameter: 3 cm and height: 5 cm) filled with oil (*σ* of 0.32 S/m and *ε*_r_ of 2.8) was inserted in the phantom from the inferior (neck) end of the phantom for the reference of isocenter in the phantom [48].

All MR experiments were performed using a 10.5 T/88 cm whole body magnet (Agilent, Santa Clara, CA, USA) interfaced with a Siemens MAGNETOM 10.5 T console (Siemens, Erlangen, Germany), supporting up to 16-channel parallel transmitters and 32-channel independent receivers. The 32-channel loop and sleeve antenna arrays were connected into the MRI system table with four Siemens TIM plugs each containing 8 receiver connections and the associated detuning signal lines.

**Fig. 3.**
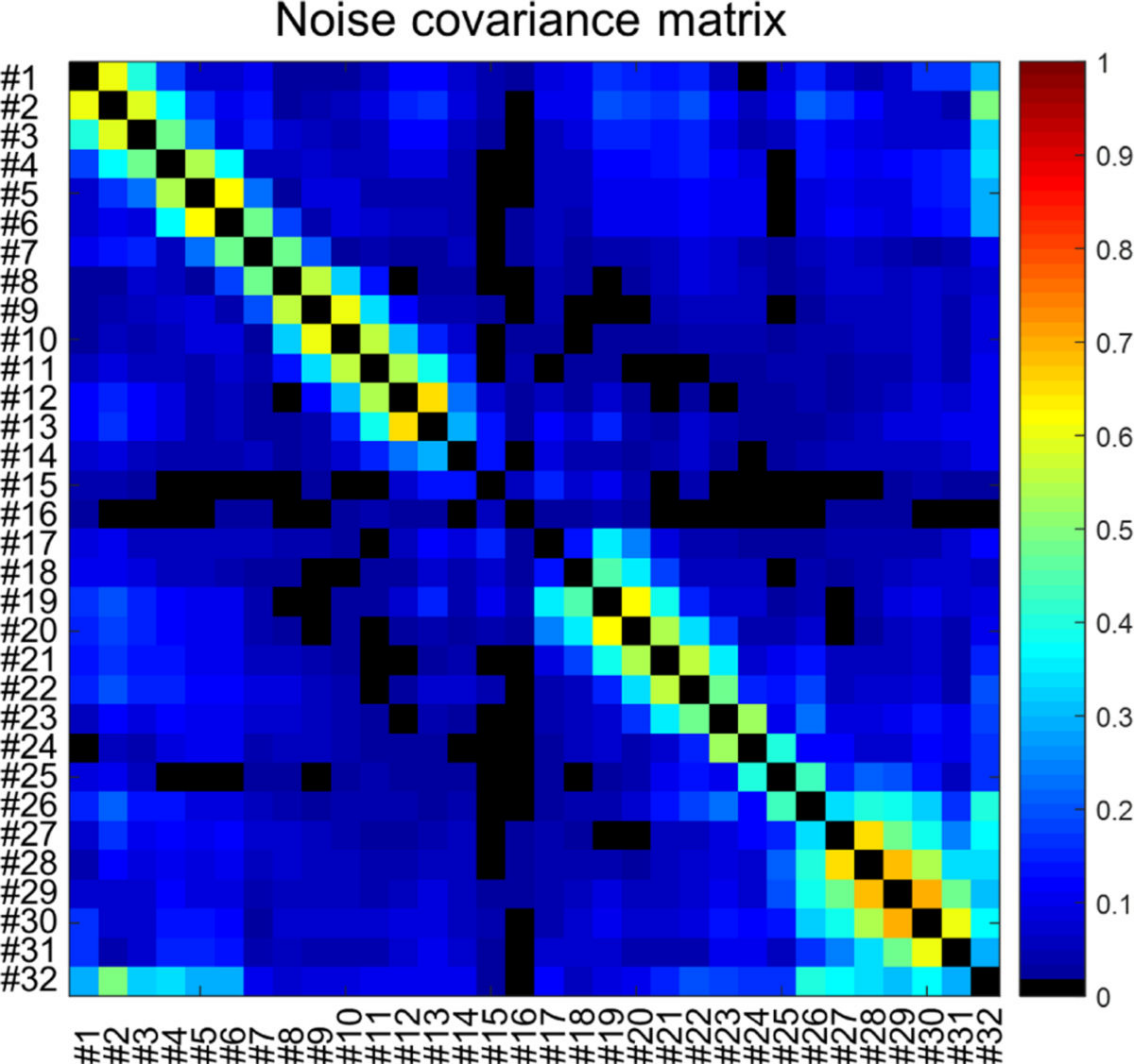
Noise covariance matrix of the 32-channel sleeve antenna array. Five inductor-shortened channels (from channel #14 to #18) on the anterior side show lower covariance values compared to the other 27 channels.

**Fig. 4.**
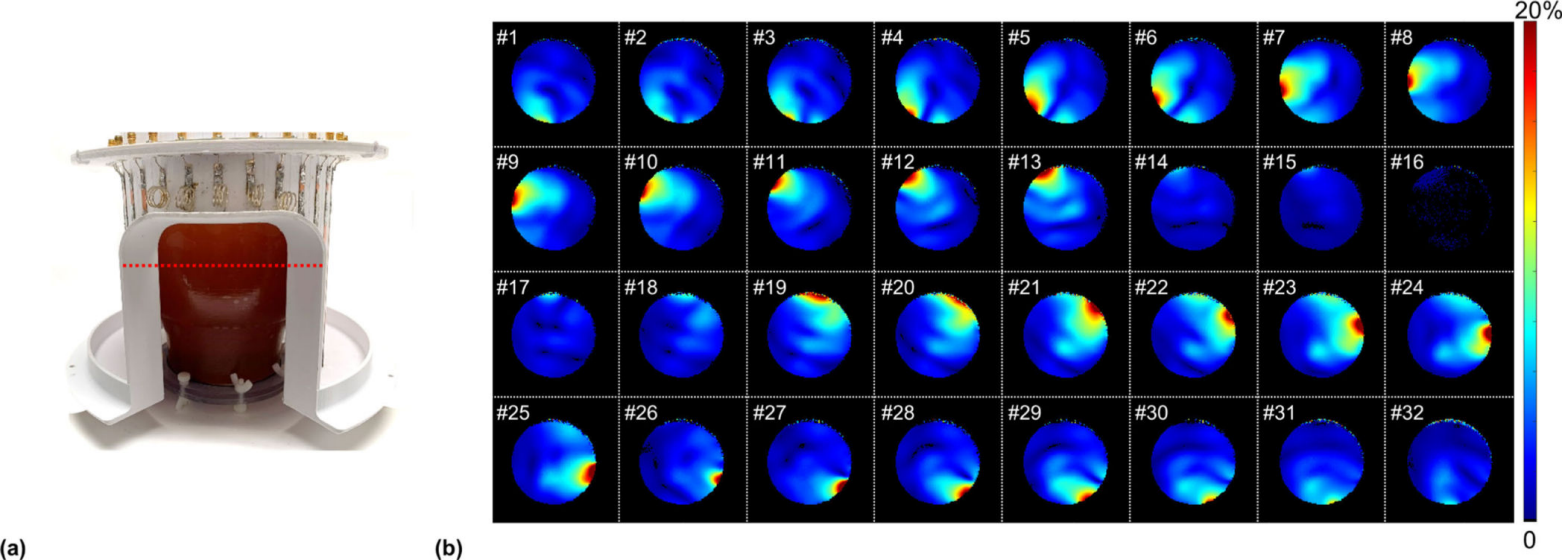
The picture of the 32-channel sleeve antenna array with the human head shaped phantom (a) and individual relative receive fields of each channel (b). The inductor-shortened 5-channels (from channel #14 to #18) for the face opening contribute less compared to the other 27-channels. In Fig. 4a, a red dotted line which is located in the isocenter of the phantom indicate the location of the individual receive field maps displayed in Fig. 4b.

The noise covariance matrix of the 32-channel sleeve antenna array (Fig. 3) was acquired experimentally to evaluate the crosstalk between the channels [49]. In Fig. 4, relative individual receive fields of all sleeve array channels are displayed which were acquired to evaluate the performance of each channel in the presence of a human head shape phantom. Each individual magnitude receive map is divided by the square root sum of squares of all thirty-two individual receiver maps.

**Fig. 5.**
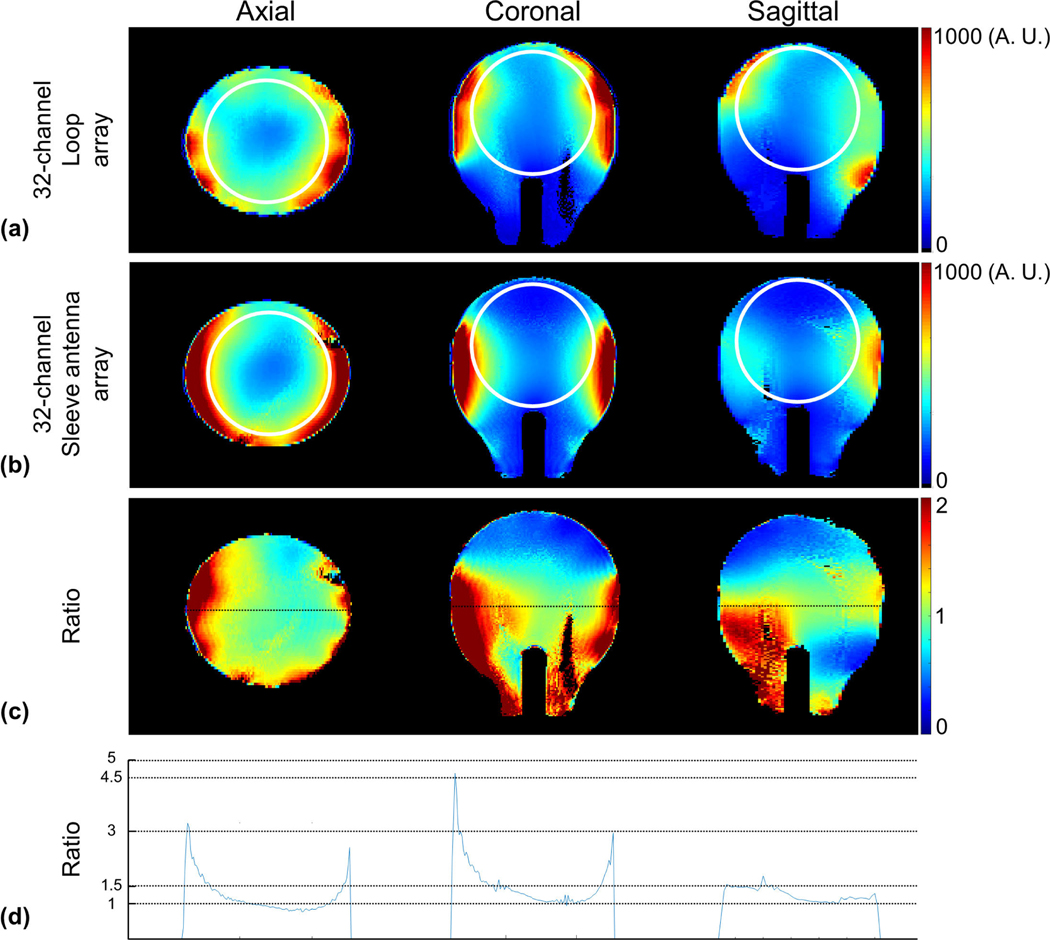
iSNR maps of the 32-channel loop (a) and sleeve antenna (b) arrays with the phantom in the axial, coronal, and sagittal planes. Ratio maps (c) between (a) and (b). The profiles (d) were obtained from the location of black dotted lines of ratio maps (c). In Fig. 5a and 5b, each representative SNR from a central location of each coil is presented with a white circle in the phantom.

A proton-density gradient echo (GRE) sequence (TR: 4000 ms, TE: 3 ms, TA: 7:48 ms, nominal flip angle: 60°, FOV: 354 × 354, and resolution: 1.5 mm × 1.5 mm × 3 mm) was obtained to calculate the iSNR (Fig. 5) with the human shaped phantom at the isocenter of the magnet. To obtain g-factor maps, a GRE sequence (TR: 130 ms, TE: 4.1 ms, TA: 4 min 26 s, nominal flip angle: 50°, BW: 255 Hz/pixel, FOV: 256 × 256, and resolution: 0.5 mm × 0.5 mm × 5 mm) was utilized in the axial, coronal, and sagittal planes. The center slice of each orientation was used to calculate g-factor maps based on Pruessmann et al. [10]. The g-factor maps for five different acceleration factors were calculated (1 × 2, 1 × 3, 2 × 2, 2 × 3, and 3 × 3), the mean and maximum g-factor values were reported for each case in Fig. 7.

### D. Numerical Analysis and Simulation

The experimental iSNR maps were calculated with the 32-channel loop (Fig. 5a) and sleeve antenna (Fig. 5b) arrays using MATLAB (The Mathworks, Inc., Natick, MA, USA). To calculate the SNR that would be produced by a homogenous 90 degree excitation, the iSNR maps were calculated from the GRE images by reconstructing the relaxed images in SNR units and correcting for locally varying excitation as measured by a flip angle map [49], [50]. For quantitative comparisons, the ratio of SNR of the 32-channel loop array over the 32-channel sleeve antenna array were calculated by MATLAB in Fig. 5c. The profiles of the ratio at the isocenter were drawn in Fig. 5d. FSL (FMRIB Software Library, Oxford, UK) was used for the proper registration.

**Fig. 6.**
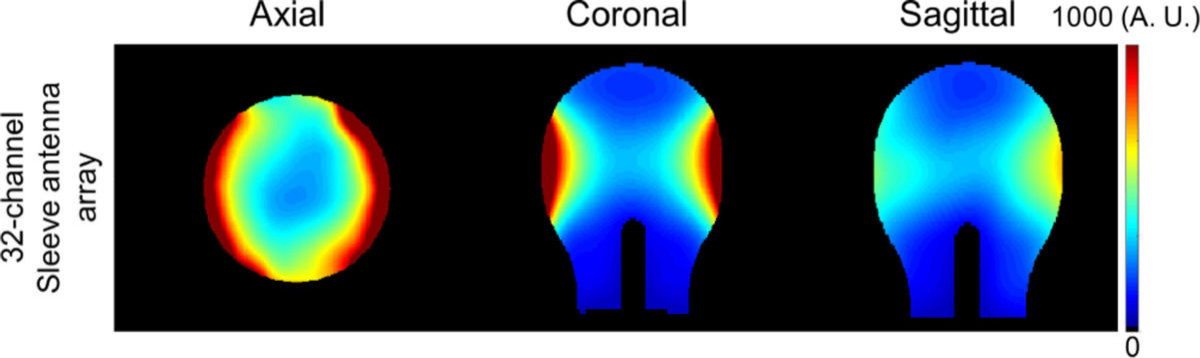
Simulated iSNR maps of the 32-channel sleeve antenna array with phantom in the axial, coronal, and sagittal planes.

With electromagnetic (EM) simulation (XFdtd, REMCOM, State College, PA, USA), B^−^_1_ fields were calculated to compare with the experimental acquired iSNR maps using the phantom with the dimensions and contents as previously described as shown in Fig. 6. All cable traps were modeled and included in the simulation to match the experimental setup as closely as possible, with realistic dimensions and electrical characteristics for copper. The coaxial cables were modeled as parallel cylindrical central bars and pipe structures. For the realistic simulation of preamplifier decoupling technique, λ/4 cables similar in location and length to the ones of the real coil were included in the simulation.

Finally, to evaluate the interaction between the 16-channel transmit array and the 32-channel receiver array, B^+^_1_ efficiency maps (Fig. 8) without and with the 32-channel receiver array were calculated. To simulate the presence of the detuned 32-channel receiver array, a short circuit between the center conductor and the ground part of the sleeve antenna array was inserted in the simulation [51]. Then, SAR efficiency without and with the receiver array was also calculated for the safety validation as shown in Fig. 9.

## III. Results

### A. Noise Covariance Matrix and Individual Receive Field Maps of the 32-Channel Sleeve Antenna Array

Fig. 3 shows the noise covariance matrix of the 32-channel sleeve antenna array. Shortened antennas (from channel #14 to #18) showed lower noise covariance values (from 0.02 to 0.13) between the nearest neighbors compared to the longer antenna elements. However, significantly higher covariance values (up to 0.69) between the nearest neighbors were shown with the longer elements due to the very tight spacing. Among the longer elements, the tighter heavily loaded elements showed less covariance values compared to the farther less loaded elements. Noise covariance values between the next nearest channels of the twelve bottom side channels (from channel #1 to #6 and from channel #26 to #32) showed relatively higher covariance (from 0.17 to 0.51) compared to the other 15-channels (from 0.09 to 0.38).

Fig. 4a shows the location of the phantom in the sleeve antenna receiver array. The red dotted line indicates the central imaging slice of the phantom. In Fig. 4b, individual receive field patterns of the sleeve antenna array are displayed for the referred central slice. For an overall head shaped phantom, the five-inductor shortened anterior (top) channels (#14 to #18) and five posterior (bottom) channels (#30 to #32 and #1 to #2) are located slightly further away compared to the remaining twenty-two channels. This explains why the five top and five bottom side of channels displayed relatively lower receive field contribution compared to the other closer channels. Particularly notable, and expected for this central slice, the shorter and farther channels (#14 to #18) did not contribute much to the entire region-of-interest (ROI) in the phantom.

### B. Comparison of SNR Maps

Fig. 5a and 5b show the experimentally obtained iSNR maps of the 32-channel loop and sleeve antenna arrays in the axial, coronal, and sagittal planes, respectively. As observed in Fig. 5a and 5c, the 32-channel loop array with close fitting loops over the superior part of the phantom displays higher iSNR performance in the superior part of the phantom compared to the 32-channel sleeve antenna array with conductors further away from the head phantom. This results in up to 50% lower iSNR with the sleeve antenna array at the top of the head. However, the 32-channel sleeve antenna array significantly outperformed the loop array in the lower and central part of the phantom. Here the sleeve antenna array shows up to 4.8-fold higher peripheral iSNR and about 15% higher iSNR in the central area of the phantom. To compare with the experimental iSNR, we include the simulation data of the 32-channel sleeve antenna array in Fig. 6.

In Fig. 5a and 5b, white lines outline the extent of an ellipsoid volume used to calculate the average SNR over a large volume. Over this volume, the loop array has an 18% SNR advantage. However, the SNR advantage of the loop array is from the superior part of the phantom.

### C. Comparison of g-Factor Maps

**Fig. 7.**
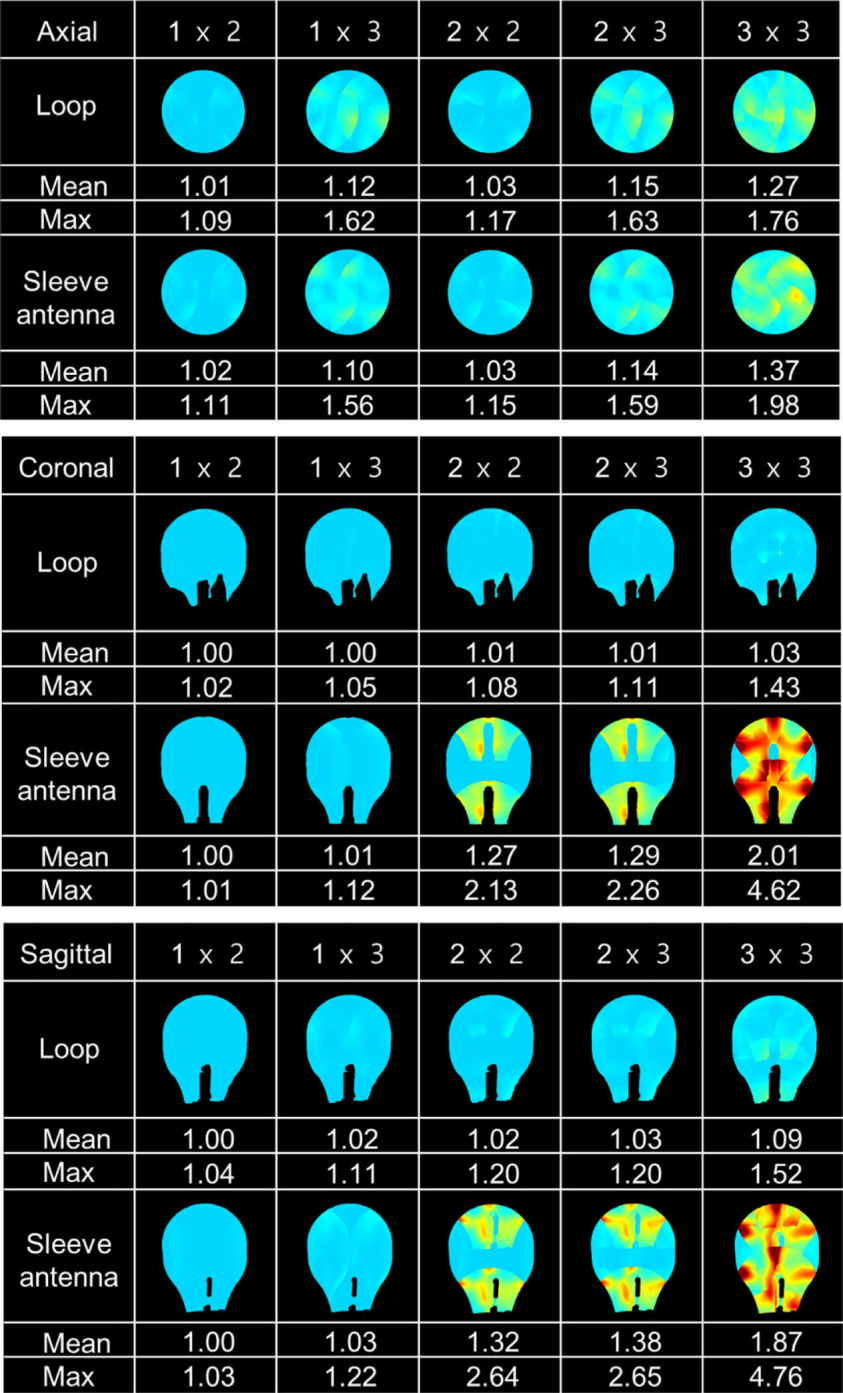
The g-factor maps of the 32-channel loop and sleeve antenna arrays with the phantom in the axial, coronal, and sagittal planes.

The g-factor maps with mean and maximum values of the 32-channel loop and sleeve antenna arrays are shown in Fig. 7. Due to the structure of the 32-channel sleeve antenna array which has a single row in the z-direction, g-factors display higher values in sagittal and coronal planes compared to the 32-channel loop array. In the transversal plane, similar g-factor are achieved for loop and sleeve antenna arrays.

### D. Comparison of B^+^_1_ and SAR Efficiency Maps Without and With the 32-Channel Sleeve Antenna Receiver Array

**Fig. 8.**
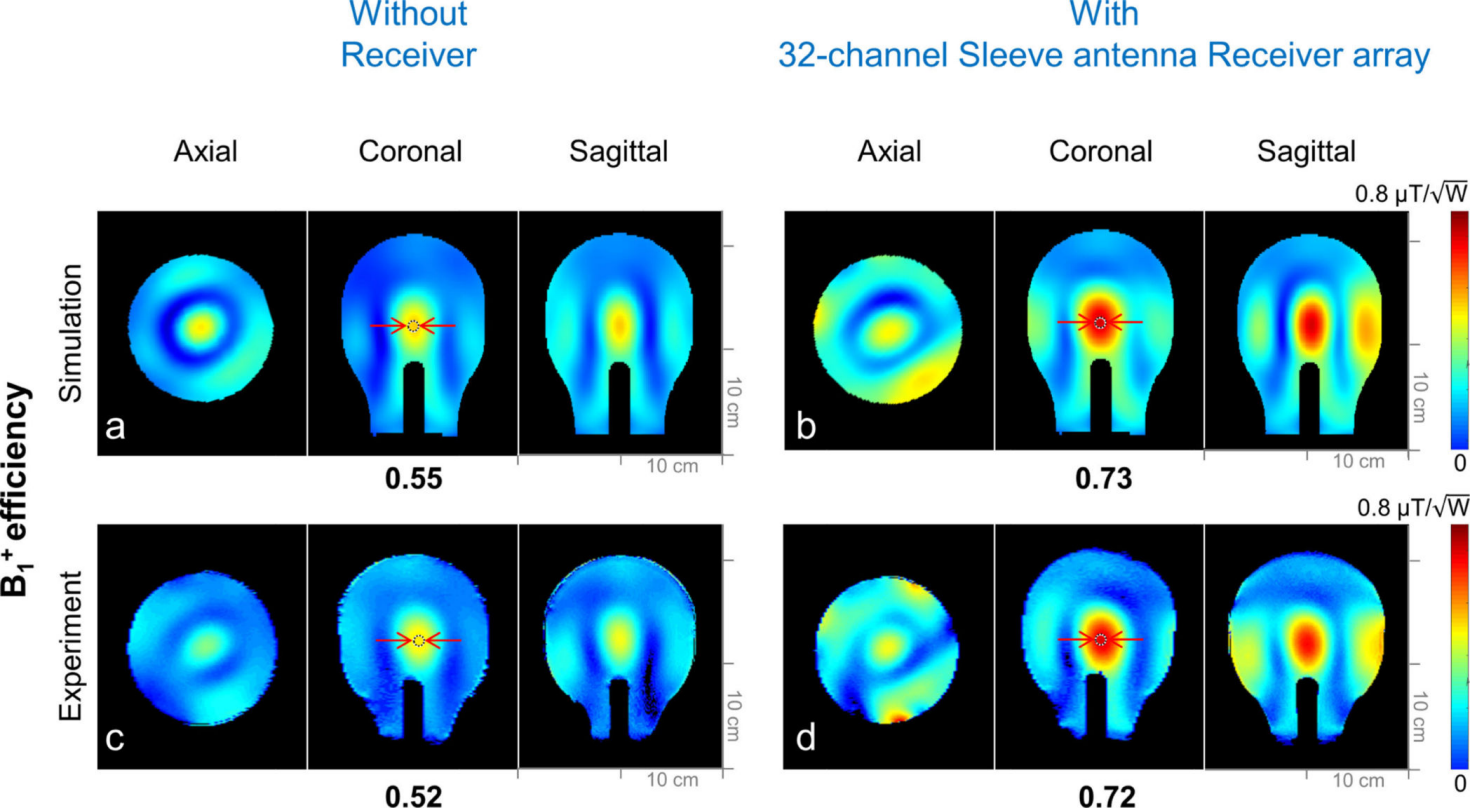
B^+^_1_ efficiency maps of the 16-channel loop array without (a and c) and with (b and d) the shortened sleeve antenna array. Red arrows indicate ROIs where values are measured and compared.

To evaluate the influence of the decoupled sleeve receiver insert on the loop transmitter array we obtained B^+^_1_ efficiency maps. The simulated and experimental B^+^_1_ efficiency maps without (Fig. 8a and 8c) and with (Fig. 8b and 8d) the 32-channel sleeve antenna array were compared in axial, coronal, and sagittal planes, respectively. Significant improvement (~24.7%) of the B^+^_1_ efficiency with the receiver array was observed both in the simulation and the experiment. Importantly for SAR evaluation we were able to consistently and accurately characterize this B^+^_1_ efficiency gain in simulation and the experiment.

**Fig. 9.**
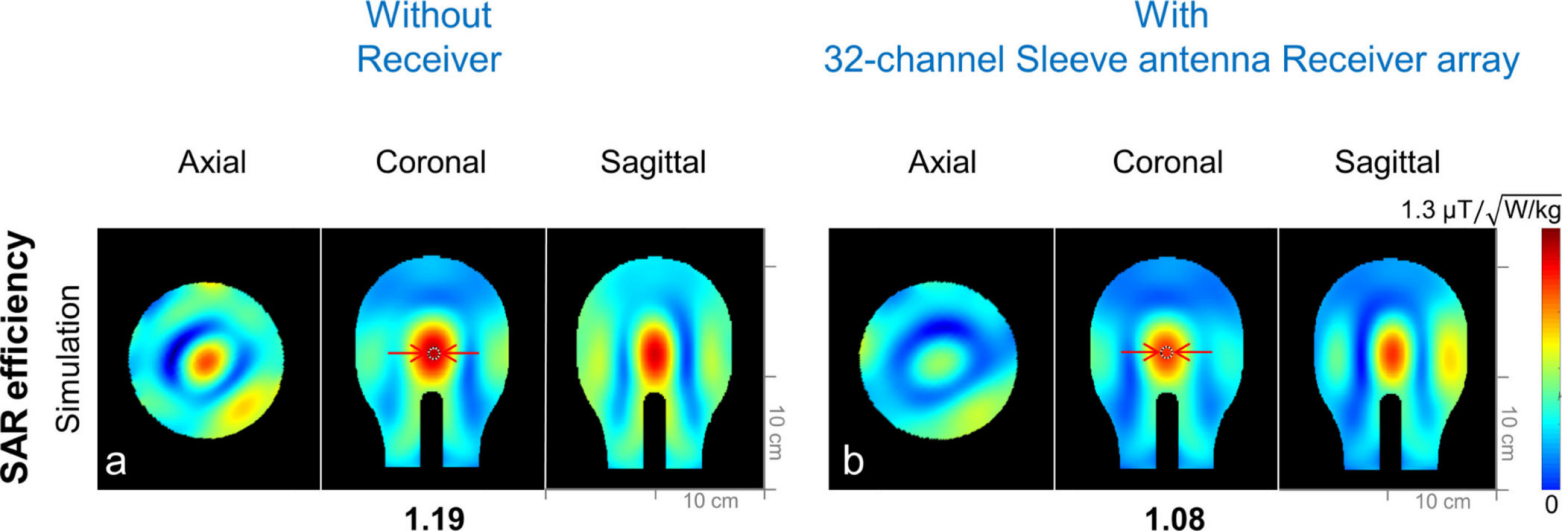
SAR efficiency maps of the 16-channel Loop Transmit array without any Receiver arrays and 16-channel Loop Transmit array with the detuned 32-channel Sleeve antenna Receiver array with phantom in the axial, coronal, and sagittal plane.

For safety validation, SAR efficiency with the receiver array is 10.2% lower compared to SAR efficiency without the receiver array as shown in Fig. 9.

## IV. Discussion

There are a number of practical hurdles when attempting to use multi-channel dipole antenna elements to construct a receive-only array. Major practical difficulties arise due to coaxial cable routing, strong coupling between channels, difficulty to detune, and the need to shorten the length of dipole antennas using lossy inductors; all of these complications can lead the substantial SNR drop [26], [52]. Sleeve antennas, which are in terms of the overall λ/2 resonance structure equivalent to the dipole antenna, have key practical advantages which can resolve some of the problems of dipoles in a dense array configuration.

Here, the main advantage of a sleeve antenna array is that it elegantly incorporates the coaxial feed cable into the antenna resonant structure, and this method can circumvent the difficult parallel alignment of one leg of a dipole with the coaxial feed cable. As a result, we observed only minimal interaction between sleeve antennas and coaxial cables. Importantly for receive array applications, this sleeve antenna array characteristic helps to significantly reduce the interaction between the coaxial output cables of the receive coil and the transmit array.

Secondly, the asymmetric structure of the sleeve antenna array and associated higher overall antenna impedance is an important steppingstone to build high channel count (≥ 32) radiative antenna arrays. This, along with preamplifier decoupling related antenna current suppression, allowed for about a 2-fold denser azimuthal sleeve antenna array compared to the dipole antenna array as shown in our previous publication [17], [40].

The comparison of the experimental iSNR between the loop and sleeve antenna arrays indicated significantly increased iSNR achievable with the sleeve array in the phantom except for the superior region of the brain. Based on Lattanzi et al. [28], the peripheral SNR is expected to dramatically increase as the numbers of loop channels are increased (The following percentage values are from Lattanzi et al). Beyond 9.4 T, however, 128-loop channels (~11%) show almost 3 to 4 times the peripheral SNR_array_/UISNR ratio compared to 32-channels of loops (~4%). However, 32-channel dipole antennas (11 to 14%) are expected to have almost similar peripheral SNR_array_/UISNR ratio compared to the 128-loop channels (~11%) above 9.4 T.

To compare with Pfrommer’s SNR ratio between Z-directed current patterns and divergence-free current patterns, we also observed a physical dimension difference between the previous theoretical modeling [29] and our work. The main difference is likely due to the difference in the overall dimension between the actual coil setup (our work) and the modeling (Pfrommer). Pfrommer et al. placed the loops on a spherical-shaped structure with 24.4 cm diameter and the dipole antennas follow the dimension of a 26 cm × 26 cm cylindrical structure. However, our design of both the 32-channel loop and sleeve antenna array follows tighter the human head shaped structure and result in an elliptical structure with a minor axis of 19 cm/18.5 cm (sleeve/loop) and major axis of 23 cm/22.5 cm (sleeve/loop), respectively. This results in the left and rightside channels of the 32-channel sleeve antenna array being located closer to the phantom than the upper and lower channels. Consequently, the side channels of the 32-channel sleeve antenna array show higher iSNR compared to the channels located on anterior and posterior sides [53], [54].

The azimuthal coil arrangement of the sleeve array supports, roughly 2.7 times higher element density in lower and central slices for the sleeve antenna array and results in substantially higher (3 to 4 times) peripheral iSNR compared to the loop array. Both experimental and simulated data supports the substantial improvement of periphery iSNR achievable with sleeve arrays at 10.5 T. However, the azimuthal alignment along with higher coupling led to reduced parallel imaging performance in axial and coronal planes compared to loop arrays.

As expected, the higher density of the sleeve antenna array led to higher noise correlation between the elements, which is due to neighboring channels of the 32-channel sleeve antenna array sharing similar ROIs and lead to higher covariance values between the adjacent channels. Overall, the noise covariance between the nearest channels of the sleeve antenna array (peak value: 0.69) is higher compared to the loop array (peak value: 0.37) and this contributed to reduced g-factors achievable with the 32-channel sleeve antenna array despite more channels in azimuthal direction. Since parallel imaging performance is essential for most UHF fMRI head applications a different alignment of sleeve antennas in combination with loops will be required in future work.

As shown in Fig. 8, the important comparison of the transmit efficiency without and with the 32-channel sleeve antenna receiver array was acquired. Significant improvement (24.7%) of the B^+^_1_ efficiency with the receiver array was observed both in the simulation and the experiment. This follows principles of deliberate coupling for improved transmit efficient described previously by Wang [55], Alipour [56], and Schmidt [57] et al.

Importantly, in terms of the safety we are able to characterize the effect of the receiver insert and calculated a 10.2% lower SAR efficiency with the sleeve receiver insert compared to without the receiver array as shown in Fig. 9. In our bench measurements, we observed detuning values with the shunt PIN diodes sufficient for preamplifier protection, however at UHF of 447 MHz the remaining conductor length of the monopole caused the observed interaction between the outer transmitter and the inner receiver.

One main reason for not adding this extra detuning circuit is that the entire performance of the radiative antenna array can be degraded by adding lumped elements. From Zhang et al. [52], the author added the PIN diode detuning circuit on each pole of the antenna for dipole receiver antennas. It is a reasonable direction which we also considered; however, with this detuning the dipole receiver array did not outperform the loop receiver array. The lumped inductors required for the detuning circuit degraded the performance of the radiative antenna array. Another reason for not adding this extra detuning circuit is that the detuning value measured with the shunt PIN diode showed fair detuning values (~20 dB) to protect the preamplifier. Perhaps one of the most exciting aspects of the proposed asymmetric sleeve antenna receive array design is that it can fully support a wearable coil concept that closely conforms to the object to be imaged. To this end, the ‘monopole’ part of the asymmetric sleeve antenna can be replaced with a fully flexible conductor (i.e. cable strands) sown into stretchable fabric. For future human head applications, the monopole side of the asymmetric sleeve antenna would be designed to be worn as a cap/hat; this concept could easily be extended to other body parts such as musculoskeletal applications with a flexible former. Such tight fitting conformal flexible coils would be excellent for peripheral SNR [53], [54] and therefore the asymmetric sleeve antenna concept has the potential to be a preferred RF coil for wearable MRI applications.

## V. Conclusion

Here we proposed a novel antenna structure for a high-density UHF MRI receiver array concept. We demonstrated results obtained with a 32-channel sleeve antenna receiver array. Our initial results indicated that the asymmetric sleeve antenna array (or arrays that use some of these concepts) can support tighter element spacing - thus enabling higher channel counts and increased antenna array element density. The results show significantly higher SNR performance of the 32-channel sleeve antenna array in the central and periphery area compared to the 32-channel loop array. The 32-channel sleeve antenna array presented here represents an initial step to demonstrate the potential of the sleeve antenna concept as receivers.

For future in-vivo UHF human brain imaging, the array will be further optimized in regard to higher channel counts and optimal lengths of the individual antennas. The high-density azimuthal sleeve antenna arrangement presented here did not support good parallel imaging performance in the axial and coronal planes. To address this shortcoming in future work, we plan to utilize a high dielectric constant (HDC) material [41] with these sleeve antennas, as well as the potential inclusion of other antenna types –such as a combination with a monopole dipole hybrid antenna array (MDH) for the improvement of the superior part of the brain [58]; we also plan to expand the overall layout towards multiple rows with the expectation of improved parallel imaging performance. Further studies should also optimize this receiver design with different types of transmit arrays.
